# “I now have a life!” Lived experiences of participation in music and theater in a mental health hospital

**DOI:** 10.1371/journal.pone.0209242

**Published:** 2018-12-18

**Authors:** Kristin Berre Ørjasæter, Larry Davidson, Marianne Hedlund, Ottar Bjerkeset, Ottar Ness

**Affiliations:** 1 Department of Social Education and Mental Health, Faculty of Nursing and Health Sciences, Nord University, Bodø, Norway; 2 Department of Public Health and Nursing, Faculty of Medicine and Health Sciences, Norwegian University of Science and Technology, Trondheim, Norway; 3 Department of Psychiatry, Program for Recovery & Community Health, Yale University School of Medicine, New Haven, Connecticut, United States of America; 4 Department of Nursing, Faculty of Nursing and Health Sciences, Nord University, Bodø, Norway; 5 Department of Social Work, Faculty of Social and Educational Science, Norwegian University of Science and Technology, Trondheim, Norway; 6 Department of Mental Health, Faculty of Medicine and Health Sciences, Norwegian University of Science and Technology, Trondheim, Norway; 7 Department of Education and lifelong Learning, Faculty of Social and Educational Science, Norwegian University of Science and Technology, Trondheim, Norway; 8 Centre for Mental Health and Substance Abuse, Faculty of Health, Social and Welfare studies, University of Southeast Norway, Drammen, Norway; University of Twente, NETHERLANDS

## Abstract

Participation in activities perceived to be meaningful is of importance in recovery processes among people with mental illness. This qualitative study explored experiences of participation in music and theater among people with long-term mental illness. Data were collected through in-depth interviews with 11 participants in a music and theater workshop carried out in a Norwegian mental health hospital context. Through a hermeneutical-phenomenological analysis, three central themes emerged: (a) engaging in the moment, (b) reclaiming everyday life, and (c) dreaming of a future. The findings indicate that participation in music and theater provided an opportunity to focus on enjoyable mundane activities and demonstrate how arts have the potential to bring meaning and more specifically small positive moments into participants’ lives. Despite seeming to be small in nature, these moments appeared to be able to add pleasure and meaning to the lives of those experiencing them. Consequently, there is a need to raise professionals’ awareness of these small positive moments of meaning, the power these experiences carry, and how to facilitate arenas which can provide such moments for people with long-term mental illness.

## Introduction

People with long-term mental illness may often experience existential frustration, wonder whether life is worth living, and strive to find meaning in their lives [1]. When struggling with mental illness, finding meaning in life is considered a central process in mental health recovery [2]. There is a hope in practice and policy considerations that participation in arts projects might have therapeutic benefits for people with mental illness [3–5] and potential to facilitate key elements in mental health recovery [6–11]. For years, mental health recovery has been seen as a unique, personal process [12], which means that each individual needs to take control of his or her life [13] and is considered the central actor in the recovery process [14–16]. But in recent years, there has been a growing understanding that recovery is more than an individual and personal process [17]. It is also seen as a social and relational process that occurs in people’s everyday lives [18, 19], in an environment imbued with meaning, in relationships with other people, and through access to meaningful activities and valued social roles [17, 20]. According to this understanding, mental health recovery is about recapturing one’s role as a healthy and contributing citizen [21, 22], with mundane everyday experiences potentially serving as building blocks in the recovery process [18, 23, 24].

It is well established that having a meaningful everyday life is important for mental health recovery [2, 25–27]. According to Thornicroft and colleagues [28], people with experiences of mental illness have stated that mental health services should place priority on meaningful activities during the day and have highly recommended future research on arts in mental health. Despite a growing interest in exploring first-person experiences related to participation in meaningful activities [23, 28–30], less attention has been paid to non-therapeutic creative activities and art programs inside the context of a mental health hospital.

In this study, we have analyzed data from a qualitative research project focusing on recovery processes among people with long-term mental illness [31]. In two previous published articles from the same study, we have focused on what enables meaningful arts participation in a mental hospital setting [31] and explored how arts participation may transform identities among people with mental illness [10]. The first article showed that continuity and flexibility in arts activities, guided by arts professionals with a non-judgment attitude, enabled participation [31]. In the second article, we found that having access to an illness-free zone where the emphasis was on the creative processes created a sense of belonging, skills-development and self-discovery which made it possible to transform previous identities [10]. In the current article, we take a broader view on arts participation and explore what experiences of participation in a music and theater workshop add to the lives of people with long-term mental illness. More specifically, we ask the following research question: What do the experiences of participating in a music and theater workshop bring to the lives of persons with long-term mental illness?

## The music and theater workshop as a research context

The music and theater workshop (MTW) is located at a Norwegian mental health hospital. It is a leisure activity not intended for therapeutic purposes. The target group is people with long-term mental illness who are in treatment or have been treated at the mental hospital as inpatients or outpatients. For some participants, the MTW also functions as a work training facility in collaboration with the Norwegian Labor and Welfare Service (NAV). A theater director works full time to run the MTW. Participation is open to all participants regardless of their psychiatric diagnosis or previous experiences with the arts.

In the MTW, participants are seen as co-creators: they are involved in all aspects of the production process, based on their interests and skills. They can participate through script writing, singing, playing instruments, creating costumes, acting, technical support, and other tasks. Further, participants are encouraged to bring their own poems, written drafts, and diary notes to the theater director. Some of these writings are transformed into dialogues or songs that become part of the MTW.

The participants in this study were offered the opportunity to collaborate with the theater director, professional actors, and musicians, both individually and in groups. Participants have weekly theater rehearsals, where they read and portray characters based on their written scripts. Through reading scripts in the rehearsals, both the theater director and the participants get ideas about how new and old scripts could function together in a play. A large music and theater production is offered annually to the public. In addition, small-scale productions take place several times a year. The MTW collaborates with the leisure center staff at the hospital, who facilitate technical, practical, and personal support, stage work, transport, and catering and have informal conversations with the participants when needed.

## Method

In this qualitative study, a hermeneutical phenomenological approach guided the research. The phenomenon *arts participation* was explored and illuminated through a phenomenological analysis inspired by Van Manen [32, 33]. Following Finlay [34, 35] and Malterud [36], we used a reflexive perspective. Through discussions among the researchers and communication of preliminary findings to involved participants, at public events, and at international research conferences, we both explored and were challenged about how our intentions and preconceptions as researchers influenced the study.

### Research ethics

The Regional Committee for Medical and Health Research (REK) in Norway approved this study (approval number: REK, 2015/476). Participants received oral and written information about the aims of the study prior to the individual qualitative interviews. An informed consent process was a requirement for participation. In the beginning of each interview the first author put a special emphasis on the right of the participants to withdraw their consent and leave the research project without having a reason or fear of negative consequences. All participants gave permission to tape-record the interviews. Because the participants were considered a vulnerable population, they had the opportunity, if needed, to speak with a psychiatrist after the research interview. The psychiatrist’s name, phone number, and email address were given to the participants in an information sheet about the study.

The consent procedures were approved by REK. All participants were considered able to consent at the time they conducted the interview, bearing in mind participants’ symptom load and general health condition [37, 38]. The first author who carried out the interview explored whether the participant understood the information provided about the study and showed the ability to understand the consequences of their own participation. In addition, the first author considered the participant`s capability to reflect on the pros and cons with participation considering own state and whether the participant had the ability to express a choice. Based on our extensive clinical and research experience in the mental health field, we were committed to the view that people with mental illness in most cases can assess themselves whether an interview is feasible [37]. However, in one case both the first author and the participant had doubt. We entered a dialogue if it would be justifiable to conduct the interview, which resulted in an agreed three months postponement of the interview.

### Participants

Participants recruited for this study had to meet the following criteria: First, they had experiences of long-term mental illness and had been an inpatient and/or outpatient in a mental health hospital. Second, participants had current or previous experiences with participation in the MTW. Third, they had been participants in the MTW for more than three months. The theater director distributed flyers to 20 current and former participants of the MTW and invited them to be part of the research project. Those who wanted to participate were encouraged to contact the first author by email, phone, or using a preaddressed envelope. Before the study started, invited participants got an opportunity to participate at an information meeting about the study, where the first author informed them about the study’s purpose and aims. In total, 11 persons participated in the study: seven women and four men, age 22 to 48 years. They had participated in the MTW from nine months to 10 years, and their contact with the mental health system ranged from three to almost 30 years. The participants themselves self-reported their health status, including self-reported diagnosis. They described experiences of psychotic symptoms, bipolar disorder, personality disorder, attention-deficit/ hyperactivity disorder (ADHD), dissociative disorder, complex post-traumatic stress disorder (PTSD), anxiety, and symptoms of depression, in addition to the use of legal and/or illegal substances to varying degrees.

### Data collection

We used in-depth interviews to collect data from participants [39, 40]. The interviews were conducted in a conversational form, based on an interview guide using open-ended questions [40] beginning with “Can you tell my about the MTW? Can you tell me about your participation in the MTW? What has participation in the MTW meant for you and your mental health?” Participants were invited to tell their stories with a minimum of interruption [41].

The majority of participants chose to be interviewed in an office located in the same building where they had their MTW rehearsals. Three participants preferred other locations for interviews: at home, in a district psychiatric center, or in a forensic hospital. Each participant was interviewed once, and the duration of the interviews ranged from 46 to 138 minutes. All interviews were carried out between June and October 2015 and were transcribed verbatim. To ensure anonymity, the participants were given pseudonyms: Anna, Benjamin, Carina, Emelin, Frida, Gabriel, Hermine, Isak, Jenny, Karoline, and Ludvik.

### Data analysis

Van Manen’s [32, 33] phenomenology of practice was used to analyze the transcribed data. According to Van Manen [33], the aim of phenomenological reflection is to grasp the essential meaning of the lived experiences of a phenomenon. In this article, we want to explore what lived experiences of the phenomenon *arts participation* brings to the lives of people with long-term mental illness. To understand how this phenomenon is experienced, with its many layers and dimensions, the thematic aspects of the lived experiences are analyzed and used as the basis for formulation of thematic phrases [33]. However, a thematic phrase will never be able to capture the full richness of the lived experience, but rather represents an aspect of the phenomenon [33, 42].

Data analysis was mainly led by the first author. To support the analysis, NVivo 11 [43] and MindManager 2017 [44] were used. These software programs were used to organize audio files and mind maps, transcribe the interviews, code the data material and write reflexive memos throughout the study. The analysis proceeded through the following steps, and will be explained below:

1. Naïve reading
2. Extracting descriptions of the lived experiences with the phenomenon from each interview transcript
3. Developing emerging themes based on all interview transcripts
4. Creating short “interpretive condensed synopses”
5. Reviewing, defining and naming themes based on discussion with participants and researchers
6. Writing up an understanding through a phenomenological reflective writing

Shortly after the completion of each interview, oral reflexive memos of the interview context, interview content and technical conducting of the interview were audio-recorded [34]. The first author did a naïve reading of the transcripts and listened carefully to the audio recordings [34, 35] to get acquainted with the data in order to get an overall impression. After the first analysis of the overall impression, the data were interpreted asking questions from the text in a curious, inquiring manner [45]. Notes on discoveries that might lead to looking at the data again were recorded. During this process, it was noted that the data contained both interesting elements related to what enabled participation [31] and what participation provided in light of the participants’ experiences. The first author went back to the audio recordings, reflexive memos, and transcripts looking for the “essence” of each interview and reflected on quotations that seemed to describe different aspects of what participation brings. Again, the analysis revealed information about participants’ experiences of arts participation in various and diverse ways. These experiences refer to both longer identity narratives [10] and shorter, more expressive descriptions showing the complexity and variety of what arts participation bring to their lives. To formulate overarching themes requires an intensive analytical process [33]. Based on analysis of all interview transcripts, three emerging themes were organized in mind maps. These were then discussed with the co-authors and used as the foundation for the further analytic process. The first author wrote short “interpretive condensed synopses” for each of the themes and discussed the findings further with the co-authors. All authors then posed questions to the proposed themes in different ways, in order to critically examine it they captured the answer to the research question in a systematic way. In addition, to critically examine the initial interpretation of the data, member checking and dialogue with other researchers about preliminary findings were used. During this process, themes were reviewed, defined and renamed. Writing is at the core of hermeneutical phenomenological analysis, where it is required that the researchers develop an ability to be sensitive to how the text “speaks about” the research phenomenon [42]. Consequently, writing has been an integral part of the ability to see and analyze the phenomenon [33]. After finalizing the analysis, the quotations used in this paper were translated from Norwegian into English.

## Findings

Three central themes emerged from the hermeneutical phenomenological analysis: (a) engaging in the moment, (b) reclaiming everyday life, and (c) dreaming of a future. However, these themes can be considered independent of each other, not always as subsequent stages.

### Engaging in the moment

The theme *engaging in the moment* suggests that having long-term mental illness can be experienced as demanding and, thus, lead to great suffering. The participants described how their mental illness took over control of their lives for longer or shorter periods of time. Some felt that it was unrealistic to completely regain control over their lives. However, they experienced the MTW as offering some respite from these problems or a space for coping, which again was central to their efforts of sustaining and fighting for a meaningful life.

Within this context, participants expressed in diverse ways how they were at one with the activity in the MTW. Jenny described how participation gave her an opportunity to focus.

“The prompter job is so wonderful. It allows me to concentrate. (…) I just shut everyone out. I exist only in my own little bubble. That bubble is only about me and what is happening onstage and who has the next line. (…) I shut everything out and become very focused on what I am doing at that point; when the lights go out, not otherwise.” (Jenny)

In line with Jenny’s experience as a prompter, Ludvik described how acting provided a mental presence of mind. Being present onstage provided a setting where he could distance himself from his problems outside the MTW. The more he immersed himself in the music or the role, the less space was available to reflect on the troublesome aspects of his life. His role demanded Ludvik to engage solely in the moment:

“It is about focusing on the present moment. (…) Here and now. Here and now. Here and now! It is about standing there, remembering lines, and not thinking, ‘Damn, I hate that I am in a mental health institution.’ Your focus is on the text. There are others onstage you need to look at. You need to go over there and wait for the heat from the light. Then you time the line over there. It is all about focusing on moments. (…) Every moment is filled with what you are doing and experiencing; that is the magic of the MTW.” (Ludvik)

Several participants described previous situations in which their mental health issues had gotten out of hand or taken over control of them. Carina expressed a powerful desire for things to be different, so she could function on a higher level. She felt trapped. Her self-destructive thoughts and actions in relation to eating and self-harm absorbed all her energy and directly affected her quality of life. Her destructiveness took up most of her time, energy, and mental space. For Carina, participation in the MTW provided a break from her self-destructive thoughts and actions. She managed to make an active choice to put her difficult life aside for a moment:

“I spend all day every day being self-destructive, except when I am here [at the MTW] Here, I am not self-destructive. The rest of the time, I am self-destructive—from morning to midnight.” (Carina)

While participation in the MTW offered a freezone, not everyone could have this experience all of the time. Some participants reported that life could be so tough at times that not even participation in music and theater gave them a break. Karoline had made a conscious decision to protect the music and theater workshop as something positive in her life. In order to maintain the positive function of the MTW, she decided to take a break from participation when things got too difficult, even though this was a hard choice to make:

“I have to take a break because it is too much right now. (…) I couldn’t even remember anything from the performance. Felt like it turned out really bad. (…) It is so important that it [participation in the MTW] remains something positive, because that is what it has been for me so far. Right now, I am too tired and stressed out.” (Karoline)

Several participants also suggested that engaging in the MTW provided moments filled with a sense of achievement that they truly enjoyed. These moments were powerful experiences in both the present and as recalled later and provided them with energy whenever their mental illness caught up with them again. Even when Karoline took a break from the MTW, she could bring with her the positive moments she had experienced there before. She deliberately returned to these moments by watching a recording of one of her performances. For her, watching these recordings was important both for recalling the positive moments and for holding on to them.

“The sense of achievement and the joy that I felt that spring! It was one of those amazing experiences. To hold on to and enjoy each moment. It strengthened me so much. (…) I need for it to feel like it did back then.” (Karoline)

The participants entered a free zone, a place where their negative/sad thoughts or destructive behaviors received less attention. Through arts activities, energy was concentrated on other facets of their life than mental illness. They experienced that arts activities require a mental presence. If the participants could not manage this presence, they could decide to take a break. They seemed to have a reflective awareness of their own participation, even in periods when they had challenges in taking part. In these periods, they could reflect on what they had been part of and what they had managed through their participation.

### Reclaiming everyday life

Participants emphasized that engagement in the MTW initiated a process through which they could reclaim everyday life. They described how long-term mental illness affected their ability to structure daily life. They stressed the importance of hanging on to everyday life routines that were meaningful. Participation in the MTW enabled the participants to regain meaningful everyday life activities.

“It makes sense for me to experience normal days. For many years, I only had special days. Days where I was either up or down, where normal everyday life was not even a possibility. (…) It is important not to fall out of everyday life. (…) It has become a sort of everyday life, too, coming here to the MTW.” (Benjamin)

Due to mental illness, several participants found it difficult to hold on to their ordinary everyday life tasks. Their mental health issues created challenges to completing their education, keeping a job, or engaging in leisure activities. Participants described a daily life in which they were inactive, either at home or in an institution. Through participation in the MTW, they experienced more active days than before. In addition, the arts activities gave them an opportunity to add meaning to everyday life activities. Gabriel underlined this point by describing how important it was to make music, not only to stare at the wall of a long-term facility: “*There is no point in just sitting there and sleeping and eating*, *and sleeping and eating*, *you know*. *That’s not a life*! *To create something—that’s fun”* (Gabriel).

Having a daily or weekly activity was of great importance to gaining a reference point in participants’ everyday lives. Participants described engagement in the MTW as a kind of lifeline. Through music and theater, they added structure to an otherwise chaotic life. As Frida stated “*The MTW became an anchor for me*.*”* Although some participants stressed the importance of having something to hold on to in their daily life, the MTW not only allowed them to take part in an activity that structured their days. They also experienced that having a meaningful activity awoke their desire to start the day, even in periods when their mental conditions became challenging. For Benjamin, participating in the workshop was important in helping him out of severe depressive episodes. In periods when he had difficulties doing anything on his own, he highlighted the importance of attending a consistent social fellowship, such as the MTW, to share his interest with others:

“Yeah, especially for the first few years, MTW held me up. It was the only thing I could do except from lying in bed unable to do anything. (…) I remember it lifting me up, lifting me up from Wednesday to Wednesday, and then to more and more practices. Then I remember how I finally got out of the mud. It lifted me out of my depression through the hours I spent practicing every week.” (Benjamin)

Experiencing mental illness for long periods of time left participants struggling to find a sense of meaning in life: *“I feel so helpless*. *It is too much*. *(…) My entire situation feels stuck after 20 years in psychiatry” (Anna)*. Participants appreciated what the MTW added to their lives. Isak highlighted the importance of having a meaningful activity to get a sense of meaning in life: *“That’s what the MTW provides*: *meaning*! *Something to do that gives meaning*.*”* Participants considered this sense of meaning in life as a necessity for surviving long-term mental illness. Suicidal thoughts, plans, and attempts were common among the participants. Several experienced the MTW as one of the most important anchors in life, which thus represented a change from feeling unmoored. They described their participation as being crucial for their existence. Isak expressed this point the following way:

“I have felt suicidal several times, and I might have actually done something about it had it not been for my participation in the MTW. I believe, hand to my heart, I can say that without the MTW, I don’t know if I would have been alive today.” (Isak)

### Dreaming of a future

The theme *dreaming of a future* capture how a clear sense of purpose and direction in life affected the participants’ motivation to both live and discover life outside of the institution. When MTW took up more space in their lives, the participants experienced increased belief in building a meaningful life outside the mental healthcare system.

Participants described having low prospects, ambivalence, or uncertainty about what they could expect of their lives before their participation in the MTW. The limitations of what they believed possible were both external and internal: in the mental health system and inside themselves. Further, participants found these limitations to be interlinked. When health professionals did not expect them to recover, it reinforced their lack of expectations for their own future. Karoline shared one example:

“In my medical journal from when I was 21 years old, it said that I was chronically ill and treatment resistant and would have to be on anti-psychotic meds for the rest of my life. I had a long list of medications. It was considered impossible to get well from schizophrenia. That was how it was put. Of course, that is not good. It makes you think, ‘What is the point of all this?’ (Karoline)

Karoline experienced a great change in what she considered possible in life after she attended the MTW. She learned to maintain hope for a better future. She reported feeling more self-assured after experiences of achievement from standing onstage performing. Karoline made a clear distinction between her plans and her dreams. Through her participation in MTW, she dared to make room for some concrete plans. However, she still found it hard to dream outside the context of the mental healthcare system:

“Of course, you never know what the future might bring, but my plan is to give lectures, publish a book, and continue with the music and theater bit. (…) I think, MTW, but maybe: I have a dream about singing, but that is more of a dream than a plan.” (Karoline)

Several participants described the MTW as opening up the possibility for them to dream more freely. Before participating in the MTW, their dreams had mainly been framed by what the mental healthcare system deemed possible for them. Through their participation in the MTW, their dreams changed. Several participants expanded their dreams from being discharged or being less dependent on using health services to living meaningful lives in their local community independent from care from the mental healthcare system. They highlighted the importance of having people around who helped them to set short- and long-term goals in areas in life other than therapy. For Jenny, the combination of setting goals both in therapy and in the MTW, which represent different areas in life, gave her a sense of psychological flexibility. A strong focus on the MTW not only fulfilled her goals, but helped her to go beyond the dreams she used to have.

“There is no doubt that the MTW has done a lot for us and for me. I never dreamt that I could be sitting here with millions in debt, own a bus, a car, and have three children. That was so far beyond my dream, beyond what I thought was possible. My psychiatrist told me a few years back that my best-case scenario would be to live at an institutional dorm that was not manned 24 hours. That was my biggest dream. I surpassed those limits miles ago.” (Jenny)

Participation in the MTW also contributed to a new pattern of behavior among the participants. In line with increased involvement, the frequency and number of readmissions they experienced went down considerably:

“I have been admitted for three weeks in the last two years. I used to be admitted for months every year. (…) There has of course been other factors than the MTW, but the way the MTW has helped me and how much less of a burden I am to the system compared to before I started participating in the MTW. (…) It costs a little to have an activity like this. But it gives you so much more in return, both in terms of quality of life and in terms of costs.” (Benjamin)

When admissions declined and quality of life increased, participants shifted their orientation toward a life outside the mental healthcare system. They gradually started to believe that they could contribute to society. Despite the fact that they had been out of work for a very long time, participants developed the skills necessary to return to the labor market or enter it for the first time. When Gabriel developed skills in music production, he started to envision a future job in the music industry and to believe that he could give something back to society.

“I want to be a part of the MTW and work professionally with music, as a sound technician or in the studio; or sit in the studio and act as a guide for others who are in psychiatry. To sit in front of the computer, create beats, run everything, you know. I have been doing this for so many years now, so I know all the programs.” (Gabriel)

Several participants described their participation in the MTW as leading to job opportunities. For Ludvik, participation worked as a stepping-stone to the labor market after years spent in the mental healthcare system. Ludvik reported that the MTW had also given him the qualifications needed to get back to work after being in a mental health hospital. Before participating in the MTW, Isak felt that his mental illness stood in the way of his chances to gain skills as an actor. In the MTW, he found that he could use his knowledge and experience of having mental illness in his performance as an actor. Through his years acting in the MTW, Isak said he built competencies that made him a sought-after actor beyond the borders of Norway. He stressed that it was important to hold on to such opportunities when possible, because no one knows what is waiting around the next corner:

“Therefore, being an actor has given me so incredibly much. (…) It is possible to be very ill and get better and have amazing experiences. Sure, I can get ill again. I never know. Statistically, according to my diagnosis, I will get ill again, but then again, not necessarily! Think about it (…) to go from being strapped to a bed not many years ago and getting disability benefits because you’ll never work again, to playing overseas! So, everything is possible!” (Isak)

Many of the participants in the MTW were people who expected to live out their lives within the confines of the mental healthcare system. However, experiences with the MTW enabled participants to see themselves differently and helped to initiate a process of recovery. In addition to reporting fewer and shorter periods of hospitalization, they had fewer symptoms and mental problems, and several were capable of establishing a life outside the hospital in a local community. After Jenny attended the MTW for years, she gradually created a life outside the mental healthcare system. She was not an active participant in the MTW at the time she was interviewed. However, she kept on dropping by the MTW to keep in touch. During her many years of hospitalization, the MTW worked as an umbilical cord for her. She reported that the MTW helped her to believe in a life outside the institution and at a certain point, it became natural to detach herself. At the same time, she pointed to the fact that establishing a new life outside the MTW had been a demanding process:

“I now have a life! I can see that life has so much to offer. Still, I must admit that I do miss life in the institution. I really do. It scared me to begin with. Now, I understand that it is part of the process. (…) I had to leave MTW. I had to leave the safety and security because I wanted to live my own life. However, I have to go back now and again to seek some comfort and predictability. This is considered absurd in psychiatry. Life is so unpredictable out there [in society]. Here [MTW], the only thing that can happen is that someone gets sicker, but it is still very predictable. (…) I have discovered that maybe we were the normal ones in here [MTW] and the rest of the world was crazy. However, I want to live where the craziness is; I like it out there.” (Jenny)

## Discussion

The main findings in this study provide descriptions of lived experiences of participatory arts in 11 adults with mental illness, recruited from a music and theater workshop in a Norwegian mental hospital. Through the arts, many participants experienced small, positive everyday moments. Further, participants highlighted that these moments had diverse impact. The small glimpses of positive moments experienced by the participants have sparked our curiosity. In our discussion, we have chosen to look closer at these moments in relation to mental health recovery.

### Moments of flow and peak experiences

The participants described moments when their focus became sharp and experiences of a world that seemed to fade away—when nothing other than being in a creative process seemed to matter. The moments were powerful and could be related to flow experiences [46] similar to those experiences of professional artists [47, 48]. While these experiences of flow came when participants were doing arts, they reported rarely having such experiences in other life situations. They valued these moments greatly and described how they empowered them in everyday life as well as in their creative processes. Interestingly, for some of the participants, these moments lasted far beyond the actual situation they encountered. It seemed that the participants had found a way to take good care of and savior these moments. They used the moments to both pursue their artwork and as a reference point in life in general. Once the participants had experienced moments of being fully dedicated and present in a performance, they strived to experience such moments again. Some of the participants wanted to take a break from the MTW rather than perform if they failed to be present in the moment. In a way, they set an expectation for themselves, not necessarily for their performance, but for their own presence when performing.

Participants also described moments when they had exceptional experiences of joy or achievement. Some of these moments could be understood as peak experiences [49, 50]. Consistent with Maslow’s findings [49, 51], participants talked about how some of their small positive moments had the potential to totally change their images of themselves and allow them to see their situations in a new way. These moments provided them with hope that they could manage situations in an otherwise chaotic life and also helped them to stake out a direction in life [52]. Their encounter with the arts challenged rigid beliefs about what they would be able to achieve despite long-term mental illness [5, 7, 9]. Some experienced these moments as guiding stars in their lives that were important for their recovery. That flow and peak experiences had impact on how they handled their mental conditions, reinforces the value of Moran and Nemec [53] suggestion of incorporating concepts and positive indicators of wellbeing (i.e., flow) from positive psychology to contribute to achieving the full vision of recovery.

### Moments of meaning

Participants also reported that doing arts made their lives more meaningful. The arts gave them a reference point in their everyday lives. Consistent with findings from Borg and Davidson [18], participants claimed that even small reference points were of importance for them in experiencing a sense of meaning in everyday life. One rehearsal a week or some meeting points a week at the MTW could be enough. They got little glimpses of being able to hold an everyday routine if they attended an activity that they experienced as meaningful. In line with Frankl [1], participants described spontaneously experiencing meaning while performing arts—some just for a few moments and others for longer periods. We posit that some of the participants moved from living in an existential vacuum to experiencing a sense of meaning in life. The creative process became a form of “personal medicine” [30] for recovery. Being among others, performing arts brought forward an awareness of what could bring meaning in life and that they could develop skills that helped to achieve this.

Consistent with previous studies [6, 8, 9], participatory arts were also found to have potential to provide participants with experiences of meaning despite suffering. This meaning was not universal, but rather an individual meaning-making that lay in each individual situation [1]. Participants emphasized the experience of meaning as the importance of feeling they were in a recovery process. They did not feel that life always had to have meaning but stated that they could build meaning in life through experiencing glimpses of meaning and fewer periods of meaninglessness. Therefore, we found it particularly interesting that music and theater enabled the participants to have more purpose and direction in the rest of their lives [6]. As noted by Moran and Alon [54], participatory arts, facilitated as a non-therapeutic activity, offer people with mental illness an opportunity to engage and develop as equal citizens in their community. Although participatory arts can facilitate meaning and initiate recovery for some, we cannot expect all people with mental illness to find a route to recovery through participatory arts [6]. On the other hand, this knowledge may have transfer value to other activities that are conducted in the mental health system and seen as leisure activities. Other avenues of creating recovery could also be pursued through other means (e.g., sport, nature).

## Conclusion

This study suggests that people with long-term mental illness may experience participation in music and theater as an opportunity to focus on activities that have the potential to provide moments of meaning, flow and peak experiences. Although these moments appeared in glimpses, they added value and gave strength to the participants as they were able to transform these positive moments to “meaning-making” far beyond arts. They experienced arts participation as liberating. This might be of particular importance to people with long-term mental illness who otherwise live a life with severe challenges, extensive contact with mental health services, and limited access to non-therapeutic arenas that support development and growth. For the participants in the music and theater workshop, the small positive moments experienced through the arts provided hope for a better life and a belief that change could be possible, which seemed to motivate and made it possible to engage in the community in the same ways as others.

## Implications

Although the field of mental health endorses a vision of recovery, people with mental illness still have limited access to arenas which support a meaningful everyday life. There is a need to increase knowledge of the importance to gain access to arenas which have a potential to provide small moments of meaning, flow and peak experiences. Since this study shows that participation in arts can provide potential for these experiences, it becomes important to discuss how professionals can facilitate arenas that provide such moments for more people with mental illness, even in periods when they are affected by increased symptoms. Simultaneously, it could be of great importance to raise professionals’ awareness of the profound role of small moments of meaning, flow and peak experiences and further examine the power of these for people with long-term mental illness. That small positive moments may play an important role in people’s lives can encourage professionals to focus beyond the big goals and rehabilitation outcomes to become more aware of the importance of mundane non-therapeutic activities [24, 55–57].

## Supporting information

S1 Interview Guide English version. (DOCX)

S2 Interview Guide Norwegian version. (DOCX)
