# Supplementary material for: “I now have a life!” Lived experiences of participation in music and theater in a mental health hospital
Source: PLoS One. 2018 Dec 18;13(12):e0209242. doi: 10.1371/journal.pone.0209242 (PMC6298666; doi:10.1371/journal.pone.0209242)
Supplement: S1 Interview Guide — (DOCX) [file pone.0209242.s001.docx]

**S1 Interview guide – English version**

Note: The guide was used as a way to prepear for the interviews. It consits of the thoughts and questions I was interested in exploring going into the study. The themes in the guide were touched upon during the interviews, but the conversations did not follow a strict structure, rather they were lead by the informants stories.

Initial Question:

1. Can you tell me a bit about yourself?
 - Age, relational status, children, work, education.


Participation in the music & theater workshop

2. Can you tell me about the music and theater workshop?

3. Can you tell me about your participation in the music and theater workshop?
 - What has been your contribution?
   - Why did you become a participant?
   
4. What has participating in the music and theather workshop meant for you and your mental health?


Contact with the mental health services

5. Can you describe what kind of mental illness(es) you experience?
 - What age were you when the mental illness(es) / mental health problems started?
 - Diagnosis

6. What kind of support do you receive now?

7. When did you come in touch with the health services and which services have you received?
 - How have you experienced the contact with the health services?

8. How is it to live with a mental illness /diagnosis?
 - What term do you use for what therapist call mental illness?

Recovery

9. What promotes and hinders recovery for you?


10. Would you say that you are in recovery?

11. What has been important for your recovery process or what will be important to experience a recovery process?

- Which of your characteristics has been important in your recovery process?

- Which external characteristics or factors has been important in your recovery process?

- What kind of role has everyday life, family, network, professionals or others had in your recovery process?

*If the person experiences a recovery process:*

12. Which parts of your life have changed through your recovery process?

 - Social networks and relations: How will you describe your social network: (Familiy, friends, others)

- Leisure and every day life: How do you spend your spare time?

- Relief from symptoms: Mood, impulsivity, suicidal thoughts / plans, self harm etc

- Hospitalization and medicine: extent and duration

13. Have participation in the music and theater workshophad impact on your recovery process?

- If so, in what way?

- Or why not?

14. Is there something you could have done differently or have wished other could have contributed with on your way to recovery?

*If the person not yet experience being in recovery*

15. From your perspective, what would be important for people with menatal illness to enable an experience of recovery?

16. Is there anything you could have done differently or wished that others could have done for you to experience recovery?


Concluding questions:

19. How would you describe the support and treatment you have received?

20. Do you have any suggestions or experiences of services or tratments that has been useful, and that you would want more of?

21. Is there any topics we have not talked about that you consider of importance in terms of mental health, arts participation or mental health recovery?
