# Supplementary material for: “I now have a life!” Lived experiences of participation in music and theater in a mental health hospital
Source: PLoS One. 2018 Dec 18;13(12):e0209242. doi: 10.1371/journal.pone.0209242 (PMC6298666; doi:10.1371/journal.pone.0209242)
Supplement: S2 Interview Guide — (DOCX) [file pone.0209242.s002.docx]

**S2 Interview guide – Norwegian version**

Intervjuguiden ble benyttet som en forberedelse til gjennomføring av intervjuene. Guiden består av tanker og spørsmål jeg var interessert i å utforske. Selv om jeg var innom alle temaene i intervjuet, hadde ikke samtalene en streng struktur, men ble heller ledet av informantenes historier.

**Innledningsspørsmål:**

1.Kan du fortelle litt om deg selv?

- Alder, sivilstand, barn, arbeid/skolegang

**Deltakelse i musikk & teaterverksted**

2.Kan du fortelle om musikk – og teaterverkstedet «Cabaret for øyeblikket»?

3.Kan du fortelle om din deltakelse i «Musikk og teaterverkstedet»?

- Hva har du bidratt med?

- Hva var bakgrunnen for at du ble aktiv?

4.Hva har deltakelsen i musikk og teaterverkstedet betydd for deg og din psykiske helse?

**Kontakt med hjelpeapparatet**

5.Kan du beskrive hva slags psykiske helseproblemer/lidelser du opplever å ha?

- Hvor gammel var du når dine helseproblemer/lidelser startet?

- Diagnose?

6.Hvilken type hjelp får du nå?

7. Når kom du i kontakt med hjelpeapparatet, og hva slags type hjelp har du fått?

- Hvordan har du opplevd kontakten med hjelpeapparatet?

8.Hvordan er det å leve med en psykisk lidelse/diagnose?

- Hva liker du å kalle det som behandlere kaller psykiske lidelser?

**Recovery (Bedring)**

9.Hva tenker du fremmer og hemmer bedring hos deg?

10.Hvor tenker du at du selv er ifht bedring?

11.Hva har vært viktig for din bedringsprosess eller hva vil være viktig for å oppleve en bedringsprosess?

- Hvilke egenskaper hos seg selv har vært viktig ifht din bedringsprosess?

- Hvilke egenskaper eller faktorer utenfor seg selv har vært viktig i din bedringsprosess?

- Hvilken rolle har dagliglivet, familie, nettverk og de profesjonelle event.andre hatt i din bedringsprosess?

*Hvis personen opplever å være i en bedringsprosess:*

12.På hvilke områder i livet ditt mener du har endret seg gjennom din bedringsprosess?

- Sosialt nettverk og relasjoner: Hvordan vil du beskrive ditt sosiale nettverk (familie, venner, bekjente)

- Fritid / dagligliv: Hvordan tilbringer du ledig tid / fritid?

- Symptomlette: Stemningsleie, impulsivitet, selvmordstanker/planer, selvskading m.m

- Innleggelser og medisin: omfang og varighet

13.Har deltakelse i musikk- og teaterverkstedet hatt betydning for din bedring?

- I så fall på hvilken måte? Eller hvorfor ikke?

14.Er det noe du selv kunne ha gjort annerledes eller ønsket at andre har bidratt med i din prosess frem til bedring?

*Hvis personen ikke opplever å være i en bedringsprosess:*

15.Hva tenker du vil være av betydning hos mennesker med psykiske lidelser for at de skal oppleve bedring?

16.Er det noe du selv kunne gjort annerledes eller ønsket at andre kunne ha gjort for at du har opplevd recovery?

**Avslutningsspørsmål:**

17.Hvordan vil du beskrive oppfølgingen og behandlingen du har fått?

18.Har du selv forslag eller erfaringer på tilbud/behandling som har vært nyttig, og som du ønsker mer av?

19.Er det tema vi ikke har snakket om som du vurderer som sentral ifht psykisk helse, kulturdeltakelse og/eller bedring?
